# Design and Material Characterization of an Inflatable Vaginal Dilator

**DOI:** 10.3390/ma17051050

**Published:** 2024-02-24

**Authors:** Po-Han Chen, Yu Ming Li, Karcher Morris, Milan T. Makale, Jyoti Mayadev, Frank E. Talke

**Affiliations:** 1 Center for Memory and Recording Research, UC San Diego, 9500 Gilman Dr. MC 0401, La Jolla, CA 92093, USA; poc002@ucsd.edu (P.-H.C.); ymli@ucsd.edu (Y.M.L.); k6morris@ucsd.edu (K.M.); 2 Moores Cancer Center, UC San Diego, 3855 Health Sciences Dr, San Diego, CA 92037, USA; mmakale@ucsd.edu (M.T.M.); jmayadev@ucsd.edu (J.M.)

**Keywords:** inflatable vaginal dilator, silicone, hyperelastic materials, Mooney–Rivlin model, digital image correlation, finite element analysis

## Abstract

There are more than 13,000 new cases of cervical cancer each year in the United States and approximately 245,000 survivors. External beam radiation and brachytherapy are the front-line treatment modalities, and 60% of patients develop vaginal damage and constriction, i.e., stenosis of the vaginal vault, greatly impeding sexual function. The incidence of vaginal stenosis (VS) following radiotherapy (RT) for anorectal cancer is 80%. VS causes serious quality of life (QoL) and psychological issues, and while standard treatment using self-administered plastic dilators is effective, acceptance and compliance are often insufficient. Based on published patient preferences, we have pursued the design of a soft inflatable dilator for treating radiotherapy-induced vaginal stenosis (VS). The critical component of the novel device is the dilator balloon wall material, which must be compliant yet able to exert therapeutic lateral force levels. We selected a commercially available silicone elastomer and characterized its stress–strain characteristics and hyperelastic properties. These parameters were quantified using uniaxial tensile testing and digital image correlation (DIC). Dilator inflation versus internal pressure was modeled and experimentally validated in order to characterize design parameters, particularly the dilator wall thickness. Our data suggest that an inflatable silicone elastomer-based vaginal dilator warrants further development in the context of a commercially available, well-tolerated, and effective device for the graded, controlled clinical management of radiotherapy-induced VS.

## 1. Introduction

### 1.1. Medical Background

In the United States, cervical cancer is most often diagnosed in young women aged 35 to 44 years, and the 5-year survival rate is 91%, according to data from the Centers for Disease Control (CDC) and the National Cancer Institute (NCI) [1,2]. Despite this high survival rate, progressive and sexually disabling radiotherapy-induced vaginal injury is a significant medical and psychological burden to survivors and society.

Standard cervical cancer therapy consists of pelvic external beam radiotherapy concurrent with chemotherapy, typically with a median dose of 45 Gy boosted with two cervically implanted radioactive pellets, brachytherapy, and boost applications of 7 Gy each [3,4,5]. These high total doses often lead to both acute and long-term radiation damage to the vaginal wall, with resultant VS in 60% of patients [6]. Acute effects include mucosal inflammation, hyperemia, and epithelial denudation, which culminates in extensive microvascular damage and swelling [7,8,9]. Delayed reactions are those occurring after three months following radiotherapy and are marked by the deposition of collagenous scar tissue, which constricts the vaginal canal [8,9,10].

**Figure 1:**
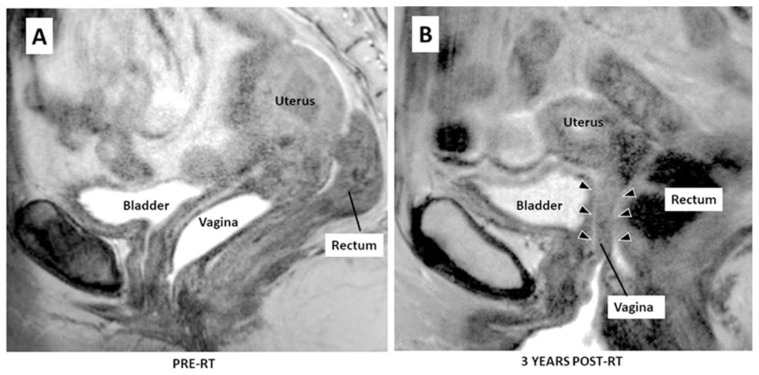
MRI image (**A**) before and (**B**) after pelvic irradiation treatment. The black arrows of (**B**) show the shorting and narrowing of the upper 2/3 of the vaginal anatomy [14].

VS develops progressively over time so that during the first year after treatment, VS is comparatively mild, but by three years post-radiotherapy, patients exhibit moderate to severe VS [11]. The salient symptoms of vaginal stenosis are shorting and narrowing of the vaginal canal and a decrease in flexibility due to the deposition of collagenous scar tissue [12,13]. In Figure 1, a typical MRI image before and after pelvic irradiation is shown [14]. VS can significantly impact cervical cancer survivors’ health and quality of life (QoL) by causing pain during medical pelvic exams, predisposition to trauma and infections, and by creating major physical impediments to sexual intercourse [15]. Approximately 60% of women who receive radiotherapy for cervical cancer go on to develop moderate to severe VS, which means that in the U.S. alone there are at least 140,000 women suffering from significant VS. Jensen et al. (2003) reported that in 45% of patients with VS, sexual intercourse could not be completed [16]. Importantly, Denton and Maher (2003) noted that clinical discussions of VS and sexual dysfunction may be constrained by age, marital status, and cultural factors. Thus, the incidence of post-irradiation therapy vaginal injury and sexual dysfunction are likely underreported [17].

### 1.2. Current Standard of Care for VS

**Figure 2:**
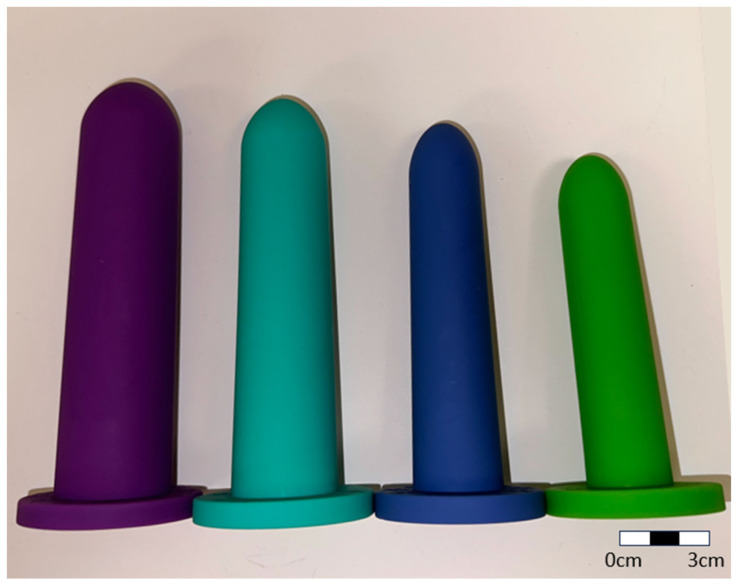
Commercially available vaginal dilators.

Hard plastic, non-personalized dilator rods are currently given to patients to self-administer, and while diligent use of these devices is effective, patient acceptance is often poor, and compliance is a major issue (see Figure 2). Most vaginal dilators presently in use are smooth plastic rods [18], as shown in Figure 2. The ill-fitting hard plastic rods being pushed at various angles into the vaginal vault are painful and this approach is disliked by many patients. Also, depending on the severity of vaginal stenosis, patients may experience difficulties transitioning to a larger dilator or finding the insertion of even the smallest size to be painful [19]. Since dilation therapy must be applied for months or years after radiation treatment, patients eventually reject dilator use because of the drawbacks and discomfort of this form of therapy [19,20].

Studies have shown that patients attribute the lack of compliance to psychological distress and physical discomfort associated with rigid dilators [7]. Patient compliance has been reported to be only 42% and to decrease over time during the duration of post-radiotherapy dilator self-treatment, leading to a noticeable change in vaginal dimensions following radiotherapy in patients who do not adhere to the prescribed dilator treatment course [20,21]. Clearly, at least for a significant proportion of cervical cancer survivors, the development of an improved dilator is a very significant medical need. Bakker et al. reported that women who were polled saw the plastic dilators as ugly and hard and preferred them to be made of soft silicone material [22].

### 1.3. An Alternative Approach

Laganà et al. (2021) and Morris et al. (2017) noted that there is an absence of appropriate management strategies for VS [7,23]. This awareness has motivated us and others to develop alternatives. Moreover, reports have indicated that patients have expressed preferences in terms of a dilator device. Patients desire a soft inflatable device. Soft inflatable medical devices have long been used by clinicians, and have important roles in the treatment of a wide range of medical conditions. For example, Pruitt and co-workers (2009) discussed the use of medical balloons for the treatment of heart disease [24], while Wu et al. (2012) designed an urethral catheter and investigated the pressure and extraction forces of an urethral catheter balloon during placement and removal [25]. Bhardwaj et al. (2018) created a balloon retractor to deform the esophagus to prevent esophageal injury during catheter ablation surgery for the treatment of atrial fibrillation [26].

### 1.4. Inflatable Vaginal Dilator Material

Our VS dilator engineering approach includes a vaginal dilator that gradually and in a controlled way expands the vaginal canal through the inflation of a balloon in order to disrupt radiation-induced collagenous scar tissue. The heart of the system is a silicone elastomer comprising the inflatable dilator walls. The dilator must be compliant in shape, soft to not damage delicate tissue, and strong to withstand pressure changes and exert sufficient lateral force against the vaginal wall to disrupt collagen scars. The addition of telemedicine components will allow physicians and biomedical engineers to keep track of prescribed dilator use, make adjustments in terms of dilator wall performance, and encourage the patient to maintain regular use in the home setting.

Polymers such as polyurethane, nylon elastomers, thermoplastic elastomers, and silicones have been found to be ideal materials for manufacturing medical balloons and soft robotic actuators [27,28]. Hsiao et al. (2019) reviewed soft robotic instruments used in surgery and found that silicone rubber is a commonly used material [27]. Asfour et al. (2020) tested medical balloons made of different materials to examine the effect of material properties on the ablation of cardiac tissue [28]. Adamson et al. (2004) developed a soft vaginal stent made of polyurethane foam to overcome the discomfort associated with rigid dilators [29].

## 2. Design and Manufacturing of an Inflatable Dilator

**Figure 3:**
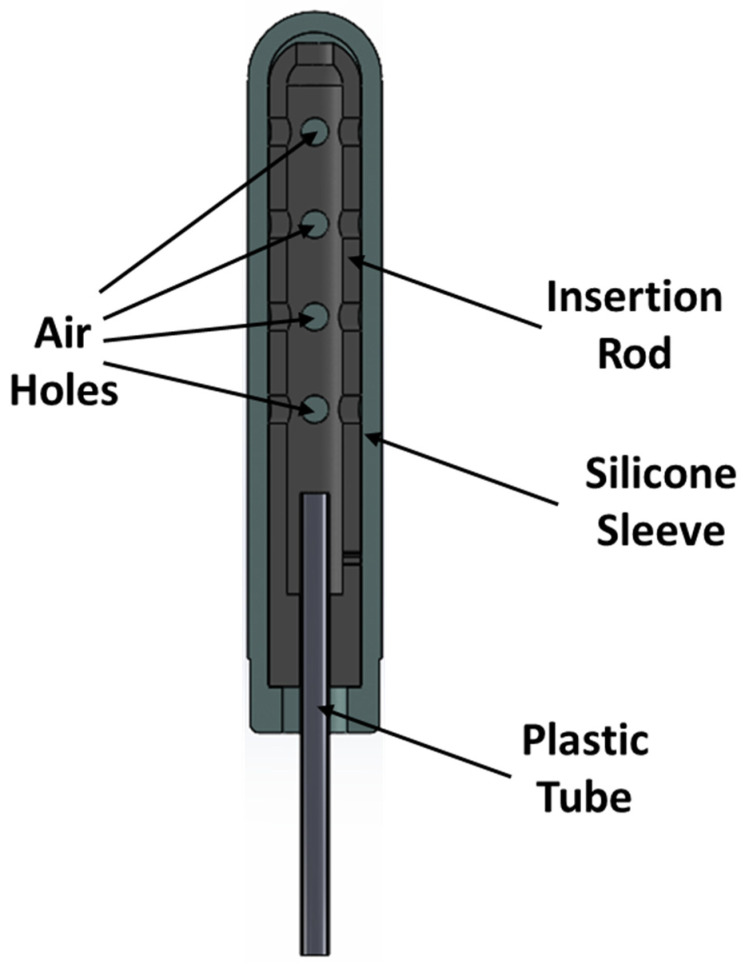
Inflatable dilator.

We show in Figure 3 a new inflatable dilator design that we have developed. The device consists of an insertion rod, a plastic tube for air supply, and an outer silicone sleeve. Air pressurizes the chamber formed between the rigid rod and the silicone sleeve during inflation, causing the silicone sleeve to expand.

**Figure 4:**
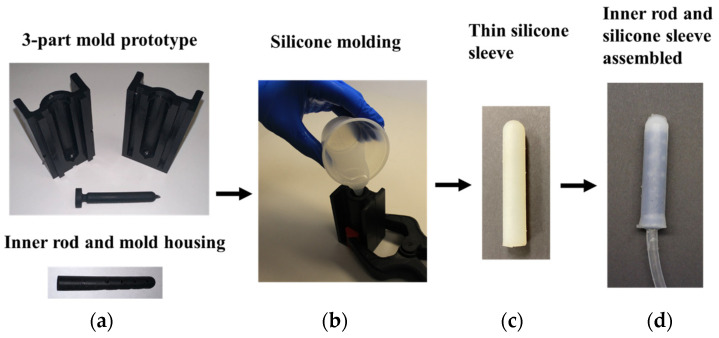
Manufacturing steps of an inflatable vaginal dilator. (**a**) 3D printed inner rod and mold housing; (**b**) molding of silicone sleeve; (**c**) thin silicone sleeve after removing from the mold; (**d**) assembled inner rod and silicone sleeve.

In Figure 4, the manufacturing process of an inflatable silicone dilator is shown, consisting of the following steps. First, a 3-part mold and an inner rod were manufactured by 3D printing (Figure 4a). Liquid silicone (Figure 4b) was then poured into the 3-part mold to form the silicone sleeve (Figure 4c). Finally, the inflatable dilator was assembled by positioning the inner rod within the silicone sleeve (Figure 4d).

## 3. Material Characterization

### 3.1. Silicone and Hyperelasticity

The required physical and material properties of medical devices directly impact design optimization. In the case of an inflatable vaginal dilator, it is clear that soft materials such as silicone elastomers must be used, and material-based characterization and optimization of the elastomers have to be performed.

**Figure 5:**
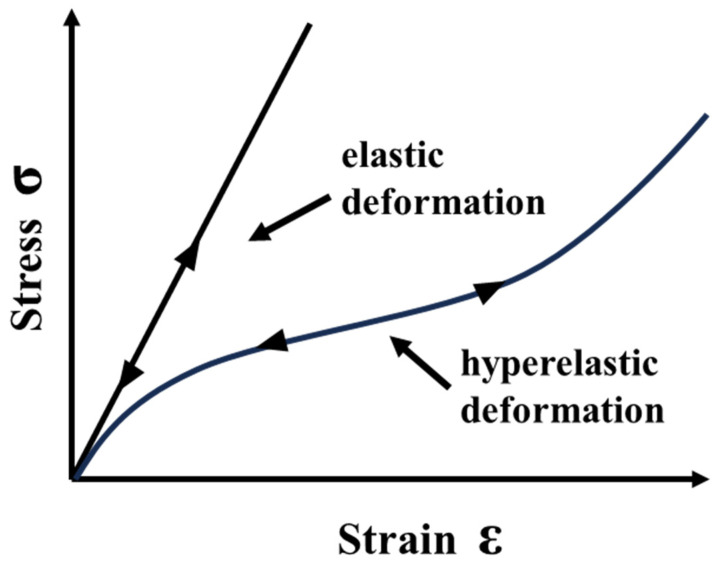
Stress–strain curve of elastic and hyperelastic material.

Polymers such as silicone are elastomers that can sustain very large elastic deformations under applied stress and reverse back to their original shape upon release of the applied stress [30,31]. Unlike plastics that deform permanently under large strain, silicone can be elongated over 100% without permanent deformation [30,32]. The silicone discussed here is classifiable as a “hyperelastic” material, that is, one that responds elastically and nonlinearly under large deformations (Figure 5) [33].

The characteristics of hyperelastic materials are that their deformation is elastic and recoverable, that their deformation is nearly incompressible, and that they show a strongly nonlinear stress–strain relationship [30,34].

### 3.2. Mooney–Rivlin Material Model

The Mooney–Rivlin material model is a widely used model to describe the stress–strain behavior of hyperelastic materials at small and medium strain levels [30,35,36]. For incompressible materials under uniform tension, the Mooney–Rivlin model can be expressed as

(1) $$\sigma =2\;C_{1}\;(\lambda^{2}-\frac{1}{\lambda })\;+\;2\;C_{2}\;(\lambda -\frac{1}{\lambda^{2}})$$

where σ is the uniaxial stress, $C_{1}$ and $C_{2}$ are material constants, λ is the normalized stretch defined as λ=1+ε, and ε is the strain [37,38].

**Figure 6:**
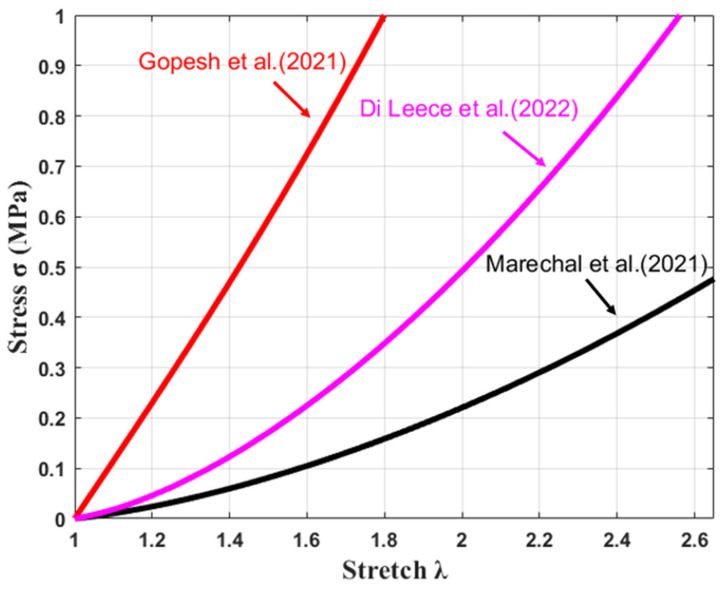
Stress–stretch plot of two-term Mooney–Rivlin curve fit [39,40,41].

Widely varying material constants $C_{1}$ and $C_{2}$ for the Mooney–Rivlin model have been published for silicone in the literature. Gopesh and Friend [39] performed biaxial testing of thin films of silicone. They determined the Mooney–Rivlin coefficients for silicone of shore hardness 10A to be $C_{1}$ = 0.18 MPa and $C_{2}$ = 0.012 MPa [39]. In a study conducted by Di Lecce et al., the coefficients $C_{1}$ = 0.12 MPa and $C_{2}$ = −0.097 MPa were found for silicone. On the other hand, the coefficients $C_{1}$ = 0.04 MPa and $C_{2}$ = −0.023 MPa were obtained by Marechal et al. (Figure 6) [40,41]. Due to the large variation in material parameters, measurements of the stress–stretch relationship for each type of silicone should be conducted before any material properties are used in numerical calculations.

### 3.3. Material Testing and Modeling

**Figure 7:**
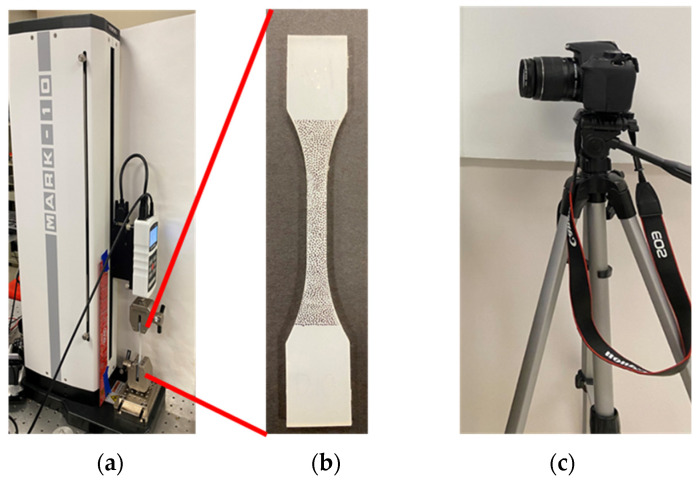
(**a**) Uniaxial tensile tester, (**b**) coupon specimen with random dot pattern, and (**c**) high-resolution digital camera.

A commercially available uniaxial tensile tester with a high-resolution camera, as shown in Figure 7a,c [30], and a coupon specimen, Figure 7b [30], were used in compliance with ASTM D412 [42]. Digital image correlation (DIC) was applied to measure the extent of stretch of the coupon specimen. Digital image correlation allowed for optical measurement of the stretch without physically contacting the sample. The coupons were speckled with a random dot pattern of 50 percent coverage, as depicted in Figure 7b [30,43]. Open-source MATLAB (R2022a) libraries, NCORR and NCORR_post, were used for sampling local stretch [30,44,45]. The stress was calculated based on the force measured from the load cell of the uniaxial tensile tester and the cross-sectional area of the coupon specimen [30].

**Figure 8:**
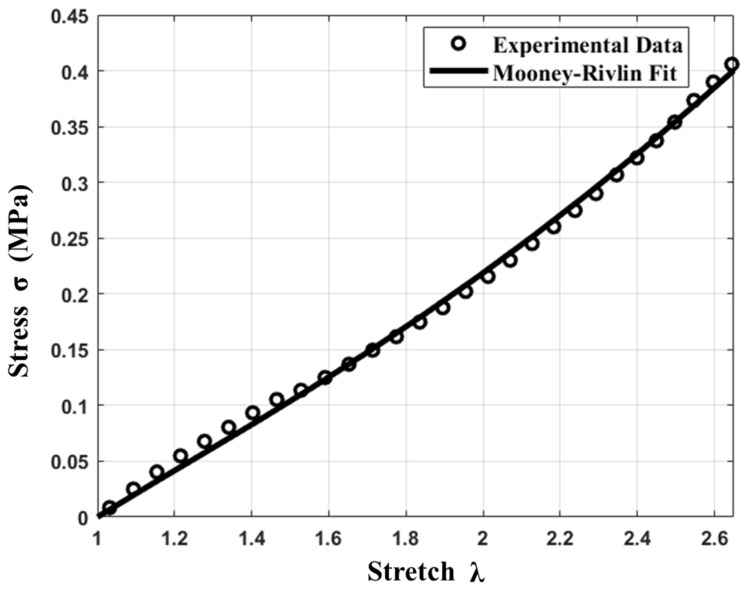
Stress–stretch experimental data and Mooney–Rivlin fit for silicone.

In Figure 8, experimental data are shown for the stress–stretch behavior of the silicone material used (Dragonskin 10 Medium, Macungie, PA, USA) for our dilator design. Least-square curve fitting yielded Mooney–Rivlin coefficients, and the solid line represents the two-parameter Mooney–Rivlin fit [30]. There was good agreement with experimental data, and the derived Mooney–Rivlin parameters were C_1_ = 0.026 MPa and C_2_ = 0.0093 MPa [30].

**Figure 9:**
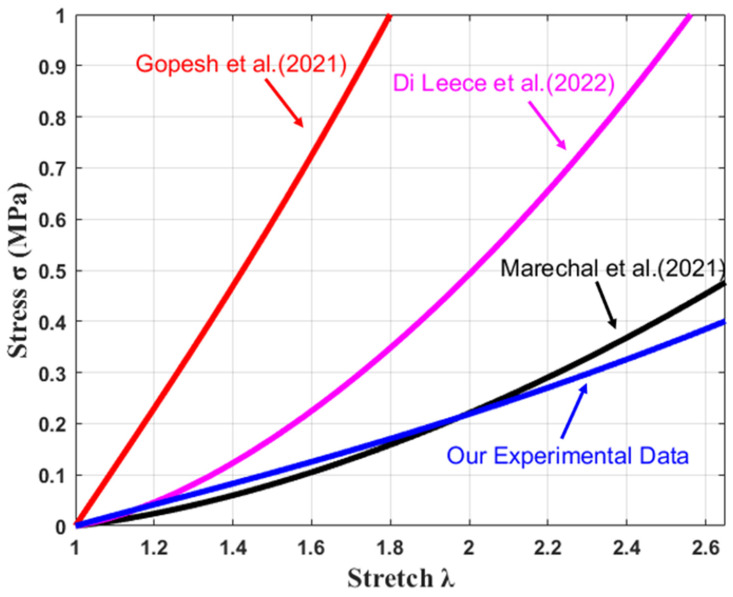
Stress–stretch plot of two-term Mooney–Rivlin curve fit (from the literature and experiment) [39,40,41].

In Figure 9, experimental data for silicone are compared to the results of Gopesh [39], Di Leece [40], and Marechal [41]. We observed that our Mooney–Rivlin parameters agree well with Marechal’s data [41]. It is likely that the difference between our Mooney–Rivlin parameters and the data of Gopesh [39] and Di Leece [40] is due to differing fabrication conditions, i.e., manufacturing temperature, curing duration, and sample thickness. In order to achieve relevant finite element simulations for our silicone, material parameters had to be acquired using samples prepared under the same manufacturing conditions as for the actual device.

## 4. Finite Element Modeling

**Figure 10:**
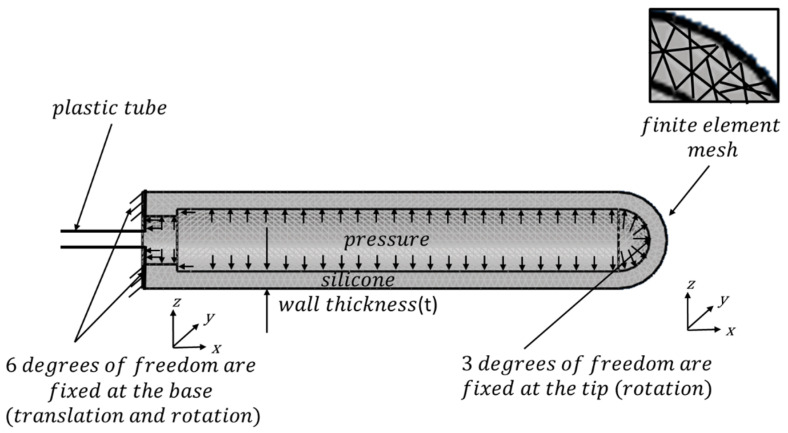
Boundary conditions of vaginal dilator for finite element analysis.

Figure 10 shows the vaginal dilator geometry and finite element boundary conditions. The dilator base was constrained to six degrees of freedom to preclude translation and rotation, and the dilator tip was limited to three degrees of freedom, thereby preventing rotation around the *X*, *Y*, and *Z* axes. Simulations were performed using finite element software (LS-Run 2022 R1), with 30,000 tetrahedral mesh elements (Altair Hypermesh 2021). Four silicone wall thicknesses, 2 mm, 2.5 mm, 3 mm, and 3.5 mm, were simulated [30].

Other dimensions, such as dilator length and outer radius, were kept constant, as were boundary conditions. The Mooney–Rivlin coefficients were C_1_ = 0.026 MPa and C_2_ = 0.0093 MPa [30].

**Figure 11:**
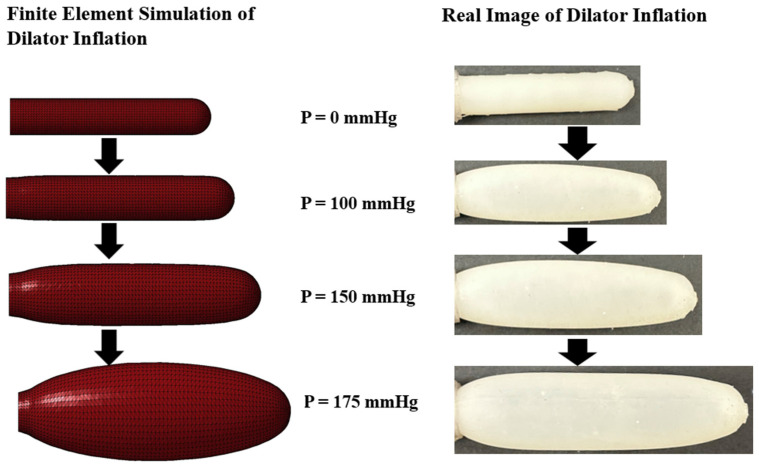
Longitudinal cross-section of dilator (2 mm wall thickness) as a function of pressure calculated using finite element analysis compared to real image of dilator.

**Figure 12:**
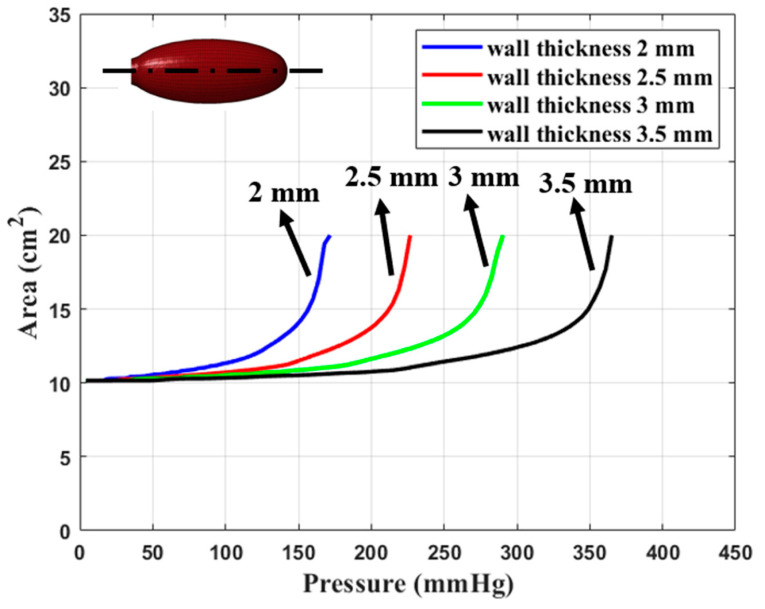
Area of longitudinal cross-section dilator as a function of pressure for wall thicknesses of 2 mm, 2.5 mm, 3 mm, and 3.5 mm, respectively (numerical results).

In Figure 11, simulation results are shown for the longitudinal cross-section of a typical dilator as a function of internal pressure, while in Figure 12, the area of the longitudinal cross-section is plotted as a function of pressure for wall thicknesses of 2 mm, 2.5 mm, 3 mm, and 3.5 mm. We observed that for any given pressure, the area of the dilator decreases with increasing wall thickness.

## 5. Experimental Verification and Comparison

**Figure 13:**
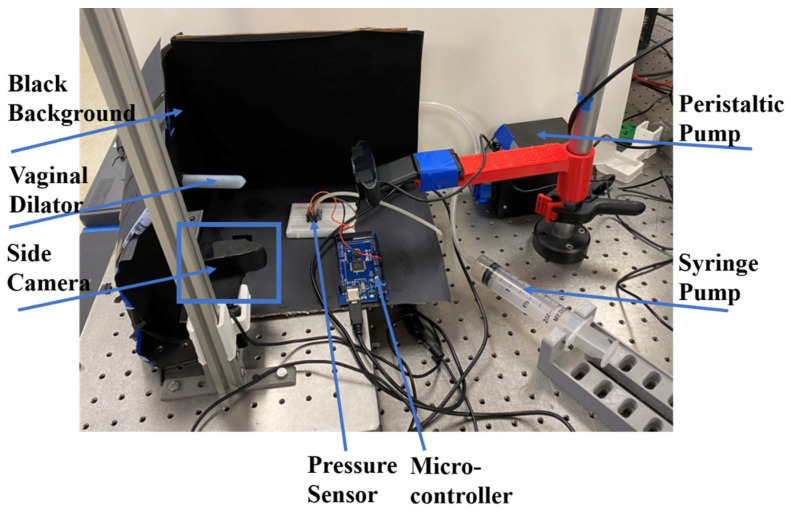
Experimental setup.

In order to compare the numerical predictions of the vaginal dilator inflation with experimental data, we measured the area of the longitudinal cross-section of the dilator as a function of internal pressure. Four wall thicknesses of dilators, viz., 2 mm, 2.5 mm, 3 mm, and 3.5 mm, were examined (Figure 13).

A high-resolution camera recorded dilator shape changes as a function of pressure, and the dilator surface area was calculated using the MATLAB image processing toolbox [30]. A black background was chosen to improve the imaging contrast of the dilator [30]. A peristaltic pump supplied a constant influx of air [30].

**Figure 14:**
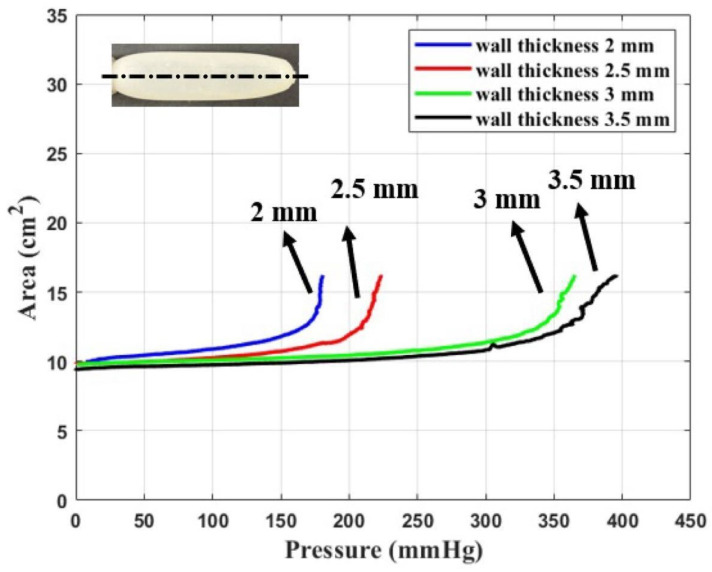
Area of longitudinal cross-section dilator versus pressure for wall thicknesses of 2 mm, 2.5 mm, 3 mm, and 3.5 mm, respectively (experimental results).

In Figure 14, the area of the longitudinal cross-section is shown as a function of pressure for wall thicknesses of 2 mm, 2.5 mm, 3 mm, and 3.5 mm. We note that an increase in wall thickness requires an increase in pressure to inflate the dilator to a specific dilator surface area value.

Comparing Figure 12 and Figure 14, we observe excellent quantitative agreement between experimental and numerical results.

## 6. Summary and Discussion

We report here on the development and testing of an inflatable cervical dilator apparatus prototype for the treatment of radiotherapy-induced VS. The proposed device is easy to use and comfortable, allowing patients to safely and effectively maintain healthy vaginal dimensions via home use. The current patient treatment model for RT-induced stenosis is the successful use of extended serial balloon dilation for radiotherapy-induced esophageal stenosis [46]. After RT to the head and neck, patients frequently develop varying degrees of fibrotic esophageal stricture [46]. Francis et al. conducted a literature meta-analysis for esophageal stenosis in patients treated with serial balloon dilations over 2 years and found excellent therapeutic results in well over 80% of the patients [47]. Similarly, our proposed research aims to develop a system for gradual vaginal expansion using a soft inflatable balloon, personalized to each patient, and immediately after RT and prior to the development of VS.

Cervical dilation has been pursued over many decades as a way of expanding the cervical vagina under various circumstances, such as childbirth and stenosis. For example, Michaels in 1980 [48] developed a cervical dilator that swells once fluid enters a flexible polymer laminate. Cowan, in 1997, [49] created a device that relied on a catheter-like balloon to expand the cervix to facilitate labor. Ochiai in 1975 [50] designed a mushroom-shaped cervical dilator to assist with birth. A major difficulty with all these devices is that they are too small in size and intended for high cervical placement, making them difficult to be positioned by the patients themselves. Crucially, they are also not designed to provide uniform and incremental pressure along the length of the vaginal vault, and they do not include feedback sensors to facilitate metered expansion of collagen scars. Hakim et al. in 2016 [51] created a detachable vaginal dilator to treat stenosis. The device was very complicated to manufacture, difficult for patients to use, and did not include an automated pump and sensor feedback system to incrementally expand the vaginal vault. The device, owing to its large dimensions, would also be difficult to insert in patients with advanced stage stenosis.

In contrast to the foregoing, the device described here is properly proportioned for ready and simple insertion, and then, once inside the vagina, is designed to be gradually widened and extended in an automated way. These features make the device easy to use and comfortable for patients. The properties of the dilator wall are critical, as the wall must safely inflate without loss of integrity, must be comfortable, conform to the vaginal shape, and must be able to exert a progressive, therapeutic level pressure on the vaginal wall. Hence, for the present study, we systematically examined the hyperelastic properties of a selected, versatile, silicone elastomer. Material properties were used to guide finite element simulations of dilator inflation under pressure.

In terms of the physical properties of the viscoelastic silicone, a very good qualitative agreement was observed between numerical predictions and experimental measurements. The material provided excellent and predictable expansion without losing integrity or forming pockets. Its deformation was reversible; in other words, it returned to its original shape and size when deflated.

A comparison of the material properties of silicone obtained in our study with values presented in the literature revealed that there are large discrepancies. These can be explained by the manner in which the silicone was processed rather than by measurement errors or variations in experimental measurement setups. Importantly, we believe that to reliably execute biomedical simulations based on silicone variants, it is essential to directly measure its hyperelastic properties, rather than simply rely on values published in the literature.

The inflatable dilator used in this study was a single-chamber design. A more advanced dilator design would be one with multiple chambers or one with variable wall thickness. In the case of multiple chambers, the pressure in each chamber could be adjusted so that the outer dimensions of the dilator change as a function of the internal pressure, allowing personalized dilator geometries based on patient-specific anatomy. In a variable wall thickness dilator, the application of a constant internal pressure would inflate regions with a smaller wall thickness to a larger diameter than regions with a larger wall thickness. This would likewise allow personalization of the dilator geometry based on patient anatomy. The case of a dilator with varying wall thickness can be formulated as an optimization problem where topology optimization together with finite element analysis is needed [52]. In addition, topology optimization can be used to reinforce the structure, resulting in a dilator with improved reliability [53].

In the studies presented in this paper, the material properties of the dilator and the design of the dilator were studied independently from the vaginal tissue surrounding the dilator during inflation. To obtain a detailed understanding of the dilator's performance during use by a patient, it will be necessary, in future studies, to include the effects of the vaginal tissue in contact with the dilator. Parameters such as contact pressure, pressure distribution, and tissue elongation need to be investigated. Again, topology optimization is a desirable method to determine the contact pressure between an inflating dilator and the vaginal tissue surrounding the dilator to determine the most patient-friendly dilation therapy [54,55,56,57].

In addition, future iterations may include surface porosity and the incorporation of chemical and biological therapeutics that could leach out and work in concert with mechanical stimulation to induce recovery of the injured vaginal wall.

## 7. Conclusions

From the investigation in this paper, we conclude the following:

1. Silicone polymers are a good material choice for use in an inflatable dilator to treat vaginal stenosis, as they are soft and reversibly deformable. The stress–stretch characteristics of a typical commercially available silicone can be described using the Mooney–Rivlin model. Large variations in material properties and Mooney–Rivlin parameters are expected depending on the manufacturing process.
2. Material properties and topology optimization of design parameters such as wall thicknesses are crucial for obtaining a well-tolerated and functional device. Reliability and manufacturability tests need to be performed to study the failure characteristics of the device.
3. Further refinements to optimally translate the dilator design into the clinic are needed, guided by close collaboration with medical professionals and, at later developmental stages, via patient feedback.
4. Medical devices such as inflatable dilators clearly have the potential to improve the lives of cervical cancer patients in multiple important ways.

## Data Availability

The data that support the findings of this study are available from the corresponding author upon request.
